# ARE FAMILY RELATIONSHIPS A PROTECTIVE RESOURCE AMONG ADULTS WITH FUNCTIONAL IMPAIRMENT?

**DOI:** 10.1093/geroni/igz038.2482

**Published:** 2019-11-08

**Authors:** Richard E Chunga, Haowei Wang, Deborah Carr

**Affiliations:** 1 University of Massachusetts Boston, Boston, Massachusetts, United States; 2 Boston University, Boston, Massachusetts, United States

## Abstract

Health-related declines that affect physical functioning are a common stressor among older adults. Functional impairment can take a toll on older adults’ psychological well-being as it limits one’s capacities to independently carry out meaningful daily activities. The extent to which impairment affects mental health may vary based on the levels of support and strain in one’s personal relationships. Stress buffering perspectives suggest that support mitigates the detrimental psychological consequences of impairment, whereas stress amplification perspectives predict that strain will amplify these consequences. We use data from 2012 and 2016 waves of the Health and Retirement Study (N=3800) to explore: (a) the direct effects of functional limitation on depressive symptoms (CES-D); (b) the extent to which these associations are moderated by spouse, child, other relative, and friend support/strain; and (c) gender and marital status differences therein. Using lagged endogenous regression models, we find that impairment significantly increases depressive symptoms among men and women, and these effects are intensified by marital strain for both married men and women. However, buffering effects are found for women only, such that marital support mitigates against depressive symptoms in the face of current impairment. These results may reflect the gendered nature of marriage, where men with impairment uniformly benefit from marriage although women may experience protective effects of only in highly supportive unions. Results for other strain and support moderators also reveal gender differences, reflecting the distinctive ways that men and women interact with kin and friends over the life course.

